# Functional expression of recombinant human trefoil factor 1 by *Escherichia coli* and *Brevibacillus choshinensis*

**DOI:** 10.1186/s12896-015-0149-5

**Published:** 2015-05-20

**Authors:** Yueh-Mei Cheng, Meng-Ting Lu, Chuan Mei Yeh

**Affiliations:** Department of Food Science and Biotechnology, National Chung-Hsing University, Taichung, Taiwan Republic of China; Agricultural Biotechnology Center, National Chung-Hsing University, Taichung, Taiwan Republic of China

**Keywords:** Glycosylated recombinant trefoil factor 1, *Escherichia coli*, *Brevibacillus choshinensis*, Secretion

## Abstract

**Background:**

Trefoil factor 1 (TFF1) mediates mucosal repair and belongs to a highly conserved trefoil factor family proteins which are secreted by epithelial cells in the stomach or colon mucous membrane. TFF1 forms a homodimer via a disulphide linkage that affects wound healing activity. Previous recombinant expressions of TFF1 were too low yield for industrial application. This study aims to improve the expression level of bioactive recombinant TFF1 (rTFF1) and facilitate application potency.

**Methods:**

The rTFF1 gene *rtff*1 was synthesized, expressed by *Escherichia coli* and secreted by *Brevibacillus choshinensis*. The rTFF1s were purified. The polymeric patterns and wound healing capacities of purified rTFF1s were checked.

**Results:**

In *Escherichia coli*, 21.08 mg/L rTFF1 was stably expressed as monomer, dimer and oligomer in soluble fraction. In *Brevebacillus choshinensis*, the rTFF1 was secreted extracellularly at high level (35.73 mg/L) and formed monomer, dimer and oligomer forms. Both proteins from different sources were purified by Ni-NTA chromatography and exhibited the wound healing activities. The rTFF1 produced by *B. choshinensis* had better wound healing capability than the rTFF1 produced by *E. co*li. After pH 2.4 buffer treatments, the purified rTFF1 formed more oligomeric forms as well as better wound healing capability. Glycosylation assay and LC-MS/MS spectrometry experiments showed that the rTFF1 produced by *B. choshinensis* was unexpectedly glycosylated at N-terminal Ser residue. The glycosylation may contribute to the better wound healing capacity.

**Conclusions:**

This study provides a potent tool of rTFF1 production to be applied in gastric damage protection and wound healing. The protein sources from *B. choshinensis* were more efficient than rTFF1 produced by *E. coli*.

**Electronic supplementary material:**

The online version of this article (doi:10.1186/s12896-015-0149-5) contains supplementary material, which is available to authorized users.

## Background

Gastric mucosal defense systems assist the gastric mucosa to withstand frequent exposure to damaging factors. Mucosal defense in the gastrointestinal tract includes local gastric mucosal defense mechanisms and neurohormonal regulation. The surface epithelial cells secrete mucus, bicarbonate and generate prostaglandins, heat shock proteins, trefoil factor peptides (TFFs), cathelicidins, and defenses to defend against or regulate mucosal damage [1]. The TFFs mediate mucosal repair by stimulating cell migration, inhibiting apoptosis and inflammation, and promoting the barrier function of mucus [2,3].

Among TFF family, trefoil factor 1 (TFF1) had been identified in human, mouse, rat and canine [4] and was secreted by epithelial cells that associated with mucus membranes of stomach. In human gastric cancer, loss of TFF1 expression was found [5,6]. The TFF1 contain 60 amino acid residues and formed a single trefoil domain. TFF1 orthologues from different species had 52% conserved in completely amino acid residues [7]. The 3-dimensional (3D) structure of TFF1 was resolved that contained 1–5, 2–4 and 3–6 disulphide bond to form a very compact structure [8]. TFF1 was predominantly expressed by gastric mucosa and co-expressed with mucins in monomer, dimer and complex forms [9]. In the adherent mucus gel layer of normal human gastric samples, the major from of TFF1 was about 25 kDa complex form, and the dimer form of TFF1 showed stronger association capability with mucins than monomer [9]. The dimer form of TFF1 had been reported to have wound healing activity *in vitro* or by *in vivo* animal models [10,11]. The structure of TFF dimeric form was determined and had significant implications for the mechanism and functional specificity of the TFF proteins [12].

Oral trefoil factors in gastric injury rats and treatment with trefoil factors in gastric cancer cell lines showed the protection against gastric damage and promoted wound healing, respectively [13]. Therefore, Production and application of TFF1 can be seen as a potent tool against injury of the gastrointestinal tract. Recombination production of TFF1 was reported in *Escherichia coli* [14], *Bacillus subtilis* system [15], and *Pichia pastoris* [16]. There was no report about yield of rTFF1 in *B. subtilis* system. In *Pichia pastoris* secretion system, the yield was about 20 mg/L but the TFF1 dimer form wasn’t observed. In *E. coli* HB10, dimer forms of TFF1 was expressed in cytosol, but the yield was low (16 mg/L) and the purification procedure was more complicated. The *B. choshinensis* was suggested to be a good candidate host to express disulfide bond containing proteins [17-19]. Characteristics such as low level of extracellular protease, high efficient secretion capability and proper folding of expressed proteins enable *B. choshinensis* expression system to be considered as a good choice for extracellular expression of disulfide bond containing proteins, such as TFF1. The secretion capacity of *B. choshinensis* was expected to simplify the purification steps to obtain rTFF1 more easily. In this study, attempts were tried to elevate expression of recombinant human TFF1 by *E. coli* cytoplasmic and *B. choshinensi* secretion expression systems. The expressions and biological activities were compared.

## Results

### Expression/secretion of recombinant TFF1s

**Figure 1:**
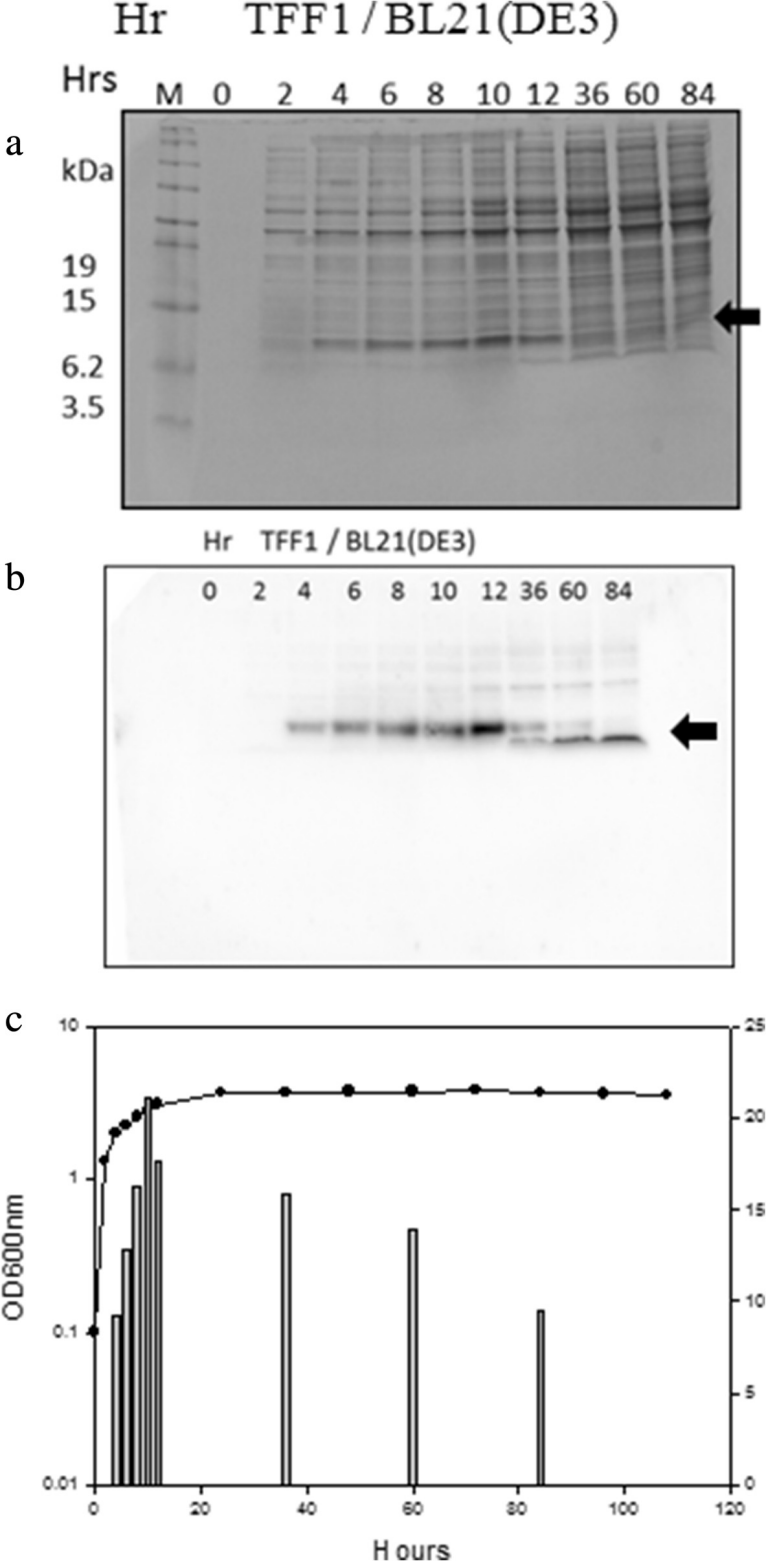
Expression of *Escherichia coli* BL21(DE3) transformants. **(a)** the SDS-PAGE analysis; **(b)** Western blot analysis; and **(c)** growth curve versus expression levels. In **(a)**, The 2 × TY cultured transformants were centrifuged, and the pellet proteins were resuspended in the same volume, loaded on gel and stained with coomassie blue. In **(b)**, the expressed supernatant proteins at various cultivation times were loaded on Tricine-SDS-PAGE, and the gel was transferred into PVDF membranes. The protein was detected with primary anti-His-Tag. **(c)**, the transformant was cultured and the OD_600_ were measured at time intervals. The productivity of rTFF1 was estimated by comparing the density of rTFF1 bands to standard rTFF1 on SDS-PAGE gels analyzed by Gel-Pro Analyzer^TM^ version 3.0 (Total-Integra Technology Co., Ltd, Taipei, Taiwan). The arrow mark indicates the rTFF1 band.

**Figure 2:**
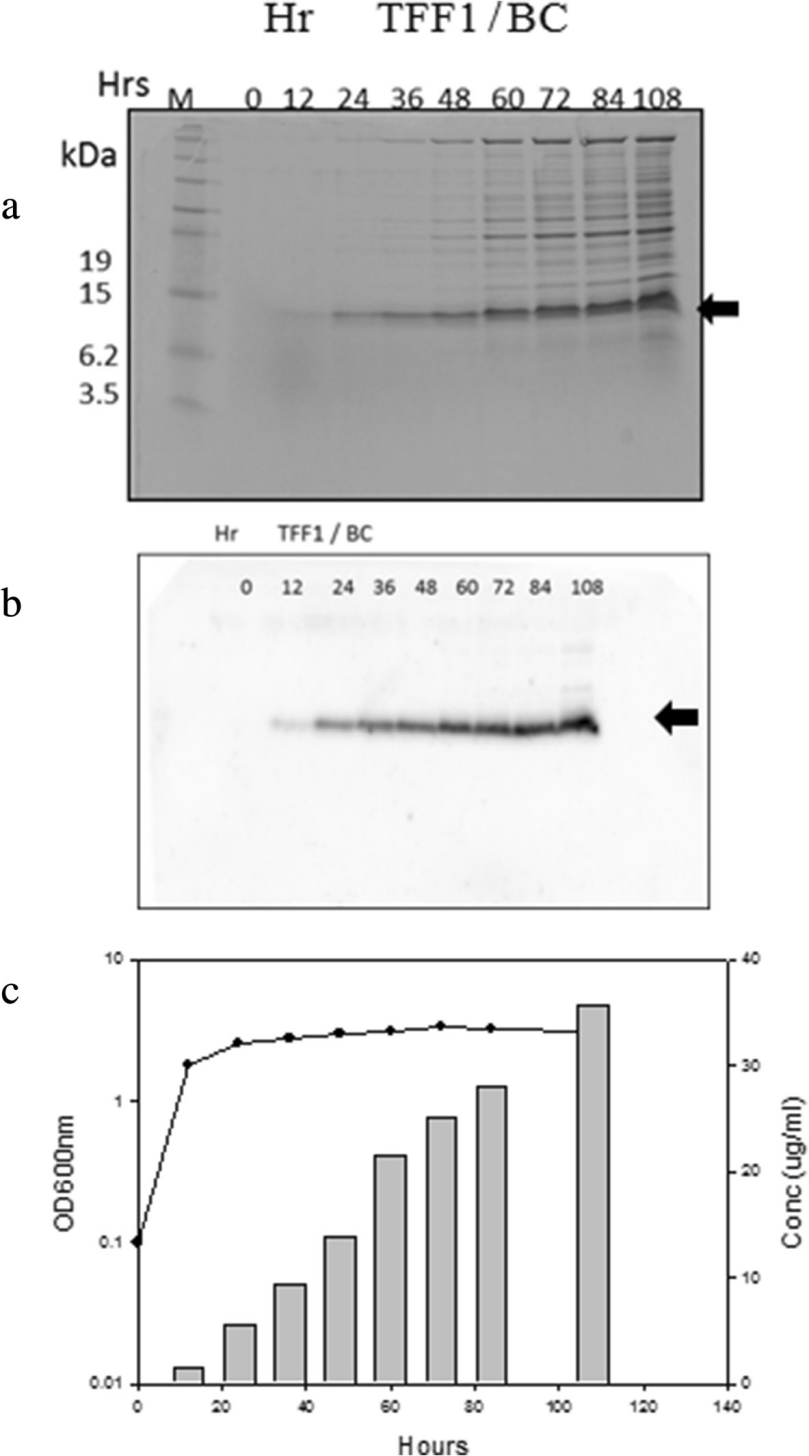
Expression of *Brevibacillus choshinensis* transformants. **(a)** SDS-PAGE analysis; **(b)** Western blot analysis; and **(c)** growth curve versus expression levels. In **(a)**, the 2SY cultured transformants were centrifuged , and the supernatant proteins were loaded on gel and stained with coomassie blue. In **(b)**, the expressed supernatant proteins at various cultivation times were loaded on Tricine-SDS-PAGE, and the gel was transferred to PVDF membranes. The protein was detected with primary anti-His-Tag. **(c)**, the transformant was cultured and the OD_600_ were measured at time intervals. The productivity of rTFF1 was estimated by comparing the density of rTFF1 bands to standard rTFF1 on SDS-PAGE gels analyzed by Gel-Pro Analyzer^TM^ version 3.0 (Total-Integra Technology Co., Ltd, Taipei, Taiwan). The arrow mark indicates the rTFF1 band.

The transformant *E. coli* BL21(DE3) (pET-TFF1) expressed rTFF1 in cytosol by IPTG, inducing the T7 promoter. The optimal expression was 21.08 mg/L at 12 h cultivation (Figure 1). The rTFF1 was stably expressed for 10 h after inducer (IPTG) was added. After the 10 h culture time, the more inducing time the more degradation was observed (Figure 1b). In the *B. choshinensis* system, transformant *B. choshinensis* (pNCMO2-TFF1) expressed rTFF1 extracellularly by using the constitutive P2 promoter and SP_Sec_ signal peptide. TFF1 continued to secrete and stably expressed until 5 days (Figure 2). The maximal expression of rTFF1 was 35.73 mg/L at time 108 h (Figure 2).

### Purification and homodimerization analysis of rTFF1

**Figure 3:**
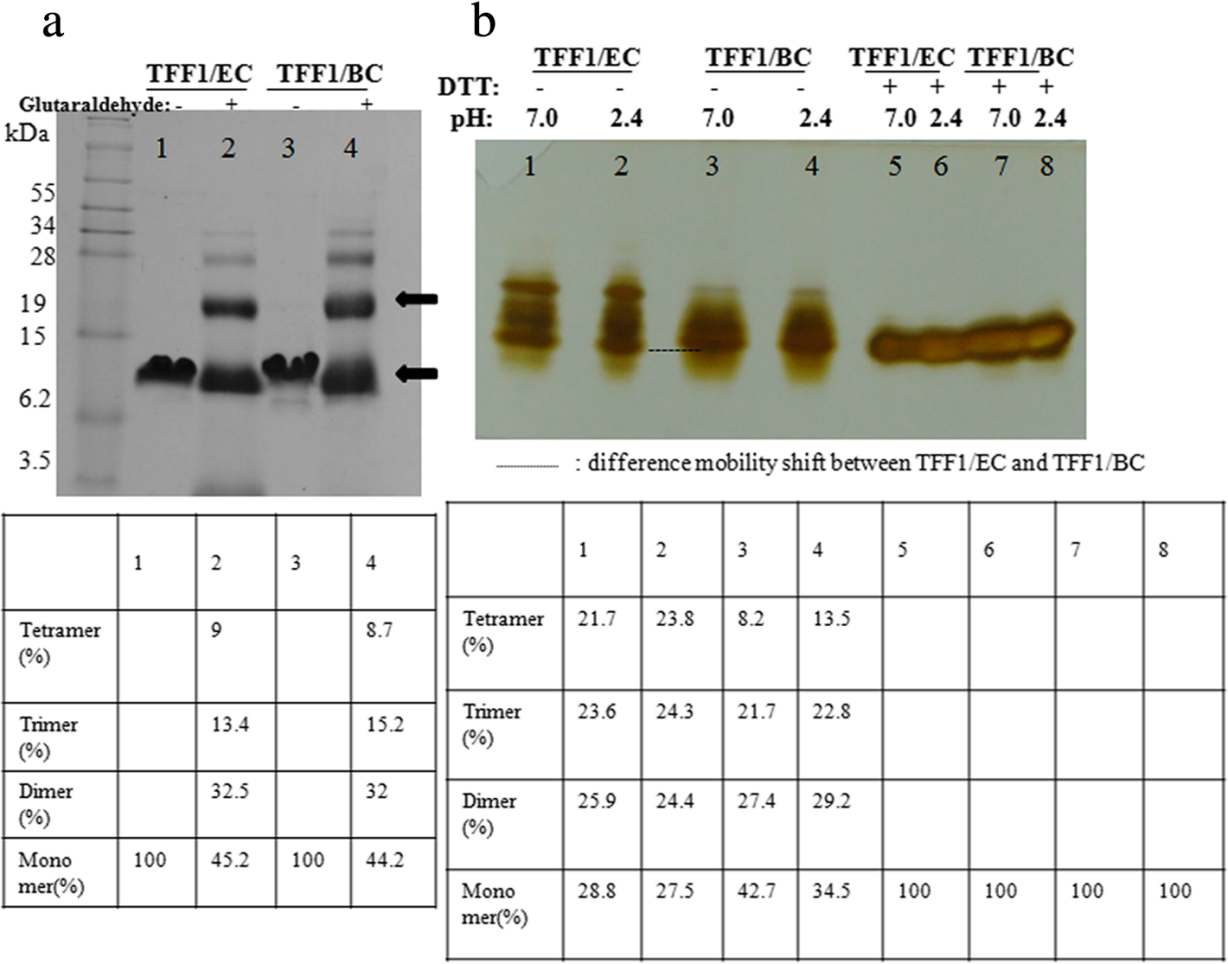
The polymerization analysis of rTFF1 purified from *B. choshinensis* (pNCMO2-TFF1) or *E. coli* BL21(DE3) (pET-TFF1). **(a)** SDS-PAGE; and **(b)** Native PAGE. In **(a)**, All glutaraldehyde crosslinker reaction products were subjected to SDS-PAGE analysis. The ratios of monomers, dimers, trimers, and tetramers were estimated by comparing the density of rTFF1 bands to non-treated rTFF1 as 100% by Multi Gauge version 3.0 (Fuji Photo Film Co., Ltd, Japan). The arrows indicate monomer and dimer forms. In **(b)**, the rTFF1 protein purified from *B. choshinensis* (pNCMO2-TFF1) or BL21(DE3) (pET-TFF1) by native Ni-NTA affinity chromatography was dialyzed against PBS buffer (50 mM Na_2_HPO_4_-NaH_2_PO_4_, pH 7.0) or Na_2_HPO_4_-citric acid buffer (pH 2.4). The purified rTFF1s were then subjected to 14% Native PAGE gel. The ratios of monomer, dimer, trimer, and tetramer forms of rTFF1s were estimated by comparing the density of rTFF1 bands to DTT treated band as 100% by Multi Gauge version 3.0 (Fuji Photo Film Co., Ltd, Japan). The dotted line indicates the mobility shift difference.

The rTFF1s were purified and analyzed by SDS-PAGE. To assess the existence of dimeric TFF1 in purified solution, glutaraldehyde was used as crosslinker to confirm the homodimeric form of protein. Glutaraldehyde treatment blurred the protein band of rTFF1 on Tricine-SDS-PAGE (Figure 3a). A significant fraction of the rTFF1 was seen on the gel and the molecular mass was the dimeric form of rTFF1. The conformation of trimer and tetramer was also presented. This result indicates that intermolecular cross-linking between free thiol of TFF1 may contribute to homodimer formation.

### Effect of acid treatment on Polymerization of rTFF1

To examine whether the acidic condition in the stomach affects the polymerization of rTFF1, the purified rTFF1s were treated with different buffers (pH 7.0, pH 2.4) and analyzed. Significant conformation differences were observed between rTFF1/EC and rTFF1/BC. The rTFF1 from all sources had the same monomer protein forms prior to and after pH treatment on a native gel with reducing agent (DTT). Alternatively, the rTFF1/EC exhibited more tetrameric forms on native gel without DTT. The rTFF1/BC exhibited monomeric, dimeric, and trimeric forms on native gel without DTT. Pretreatment with pH 2.4 buffer promoted more trimeric forms (Figure 3b).

To further evaluate the polymerization pattern of rTFF1s, the molecular weights of rTFF1s were determined by MALDI-TOF mass spectrometry to compare with the observed polymerization patterns on native gel. The purified rTFF1s from both sources were treated with different buffers (pH 7.0, pH 2.4) and examined. The different peak valleys were presented in buffers (pH 7.0, pH 2.4) with or without DTT treatment (Additional file 1: Figure S1). The monomer, dimer, trimer, tetramer, and pentamer polymeric forms were observed in all pH treatments’ rTFF1/EC; only monomer and dimer forms were observed after DTT treating. Similar results were observed on rTFF1/BC, but the polymerization forms of rTFF1s were monomer, dimer, trimer, and tetramer. The rTFF1/EC exhibited more polymerization capability than rTFF1/BC.

### Post-translational modification of the rTFF1 secreted by B. choshinensis

**Figure 4:**
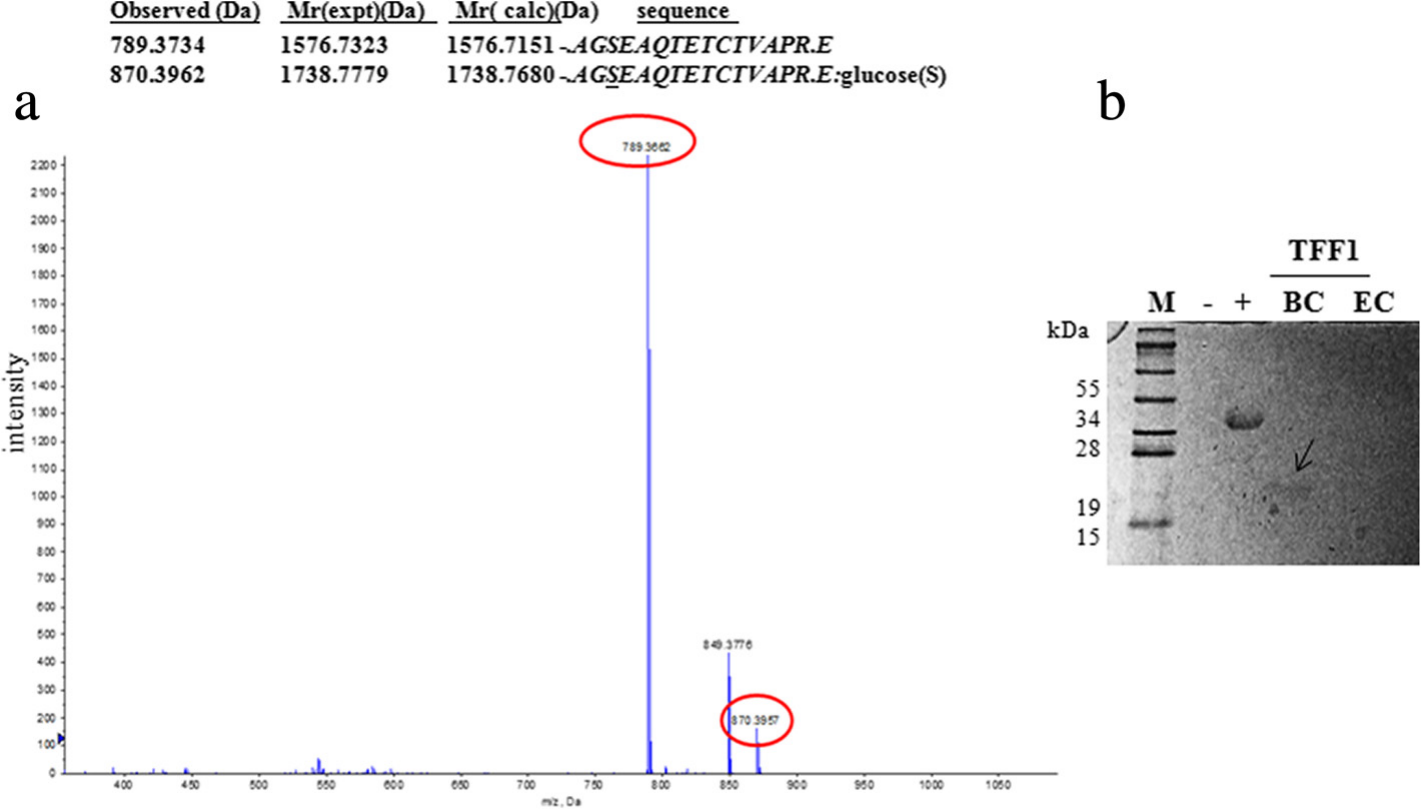
The LC-MS/MS spectrometry analysis of the extra 162 Da mass on N-terminal AGS of rTFF1. **(a)** BC (pNCMO2-TFF1); and **(b)** glycoprotein stain analysis. The rTFF1 protein purified from *B. choshinensis* (pNCMO2-TFF1) by native Ni-NTA affinity chromatography was dialyzed against PBS buffer (50 mM Na2HPO_4_-NaH2PO_4_, pH 7.0) then analyzed by LC-MS/MS spectrometry **(a)** and confirmed by glycoprotein staining method (Pierce, USA). In **(b)**, the positive control (+) was horseradish peroxidase and the negative control (−) was soybean trypsin inhibitor. The TFF1/BC and TFF1/EC represented rTFF1 proteins purified from *B. choshinensis* (pNCMO2-TFF1) and from BL21(DE3) (pET-TFF1), respectively.

The rTFF1/BC is suggested to be further modified due to two observations. First, the mobility shifts between TFF1/EC and TFF1/BC on native gel were slightly different. Second, the molecular weight of rTFF1/BC analyzed by MALDI-TOF mass spectrometry showed that an unexpected mass increase was observed. Confirmation was executed by LC-MS/MS spectrometry. An additional 162 Da mass was observed in peptide sequence **AG*S*EAQTETCTVAPR** through trypsin digestion of rTFF1. This result indicated that O-link glycosylation occurred in the N-terminal Ser residue of rTFF1 (Figure 4a). To identity modification of rTFF1, the glycoprotein sugar moieties were detected with periodic acid stain method on tricine SDS-polyacrylamide gel. The result showed that sugar moieties of rTFF1/BC were present (Figure 4b). There was no sugar moiety observed in rTFF1/EC (Figure 4b).

### The rTFF1 induced wound healing

**Figure 5:**
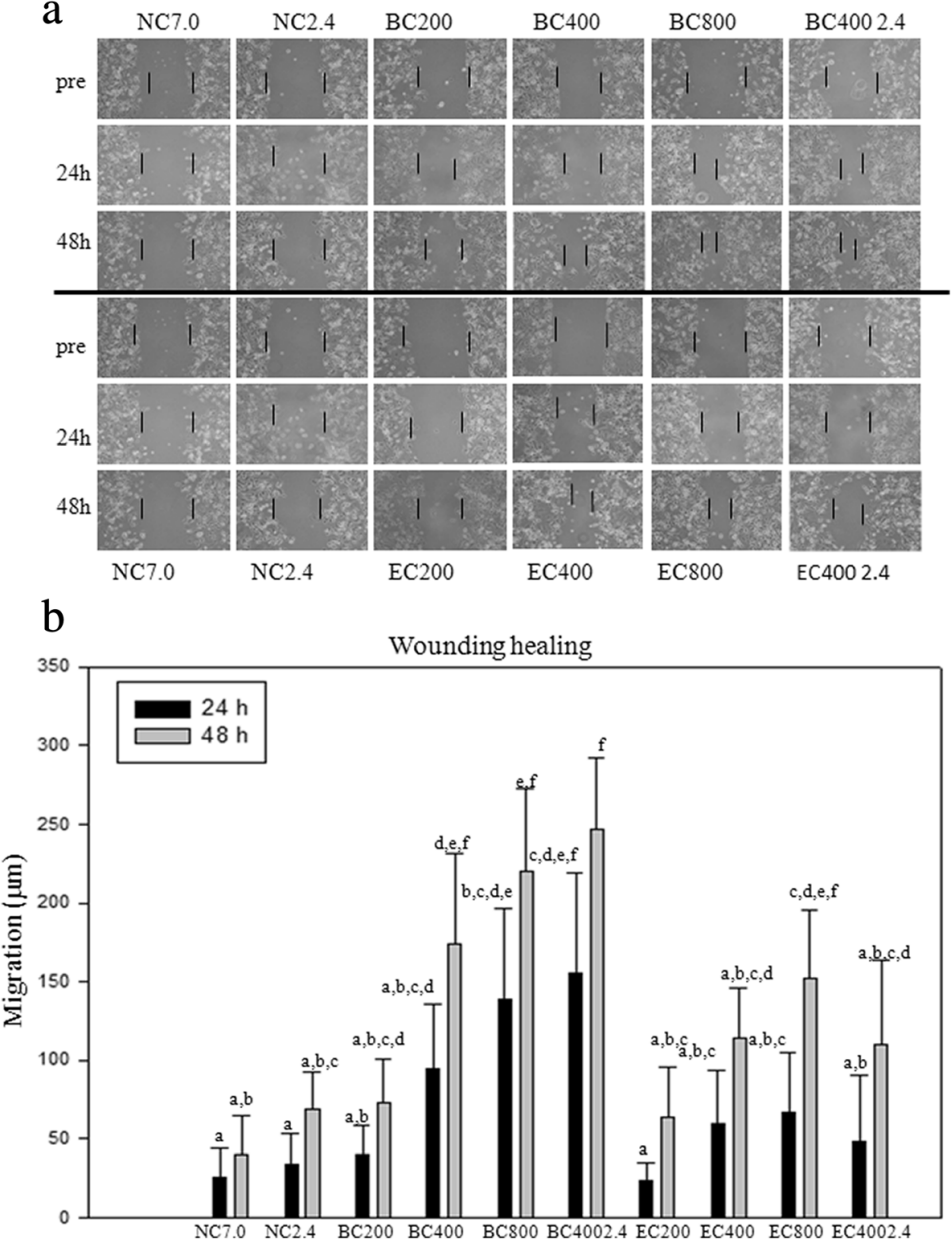
The wound-healing capacities assay. **(a)** wound-healing capacities; and **(b)** statistical analysis of the wound healing travel distances. In **(a)**, the various concentrations of rTFF1 (200 ng /mL, 400 ng/mL, 800 ng/mL) in pH 7.0 buffer and the concentration of rTFF1 (400 ng/mL) in pH 2.4 buffer were added and cultured in Ham’s F-12 K medium containing 10% FBS. The cell cultures were maintained at 37°C under an atmosphere of 5% CO_2_. The wound closure was monitored and photographed at 0, 24, and 48 h with a Nikon TS100 inverted-microscope and a Nikon D5100 camera (Nikon, Japan). BC200, BC400, and BC800 represent the rTFF1 purified from *B. choshinensis* (pNCMO2-TFF1) with concentrations of rTFF1 200 ng/mL, 400 ng/mL, and 800 ng/mL in pH 7.0 buffer and EC200, EC400, EC800 represented the rTFF1 purified from *E. coli* BL21(DE3) (pET-TFF1) with concentrations of rTFF1 200 ng/mL, 400 ng/mL, and 800 ng/mL in pH 7.0 buffer. BC400 2.4 and EC400 2.4 represented rTFF1 purified from *B. choshinensis* (pNCMO2-TFF1) or from *E. coli* BL21(DE3) (pET-TFF1) with concentration of rTFF1 400 ng/mL in pH 2.4 buffer. In (b), all experiments were repeated four times and calculated with Image J 1.46r (NIH, USA). Each each bar represents the mean ± SD. NC7.0 and NC2.4 were the negative controls. The same letter represents non-significant differences of multiple comparison.

The rTFF1/EC and rTFF1/BC were subjected to wound healing assays. AGS cells were cultured with or without rTFF1 for 24 h and 48 h to examine the migration of AGS cells. The rTFF1s of lower than 400 ng/mL concentration couldn’t significantly promote cell migration under inverted-phase microscopy (100×) observation. The minimum concentration of 400 ng/mL rTFF1 was recommended for the *in vitro* cell wound healing, and higher concentration of rTFF1 (800 ng/mL) enhanced the capability of wound healing (Figure 5).

To mimic gastric conditions, purified rTFF1s were treated with buffer (pH 2.4), and then preceded the wound healing assay on AGS cells. The results showed that rTFF1 treated with pH 2.4 buffer enhanced cell mobility, especially for rTFF1/BC (Figure 5a). Measurements were made at 100× magnification. Measurements of wound width were made at beginning of the experiment and after 24 h and 48 h. The mean traveled distances by various rTFF1 added into AGS cells were examined (Image J 1.46r, NIH, USA) and the data was statistically analyzed (Figure 5b). The effects of rTFF1s on AGS cell migration were concentration dependent. From the statistical analysis data, the rTFF1/BC showed better wound healing capability than the rTFF1/EC and the pH 2.4 treatment promoted the wound healing capability of rTFF1/BC.

### Medium optimization of rTFF1 secretion production

**Figure 6:**
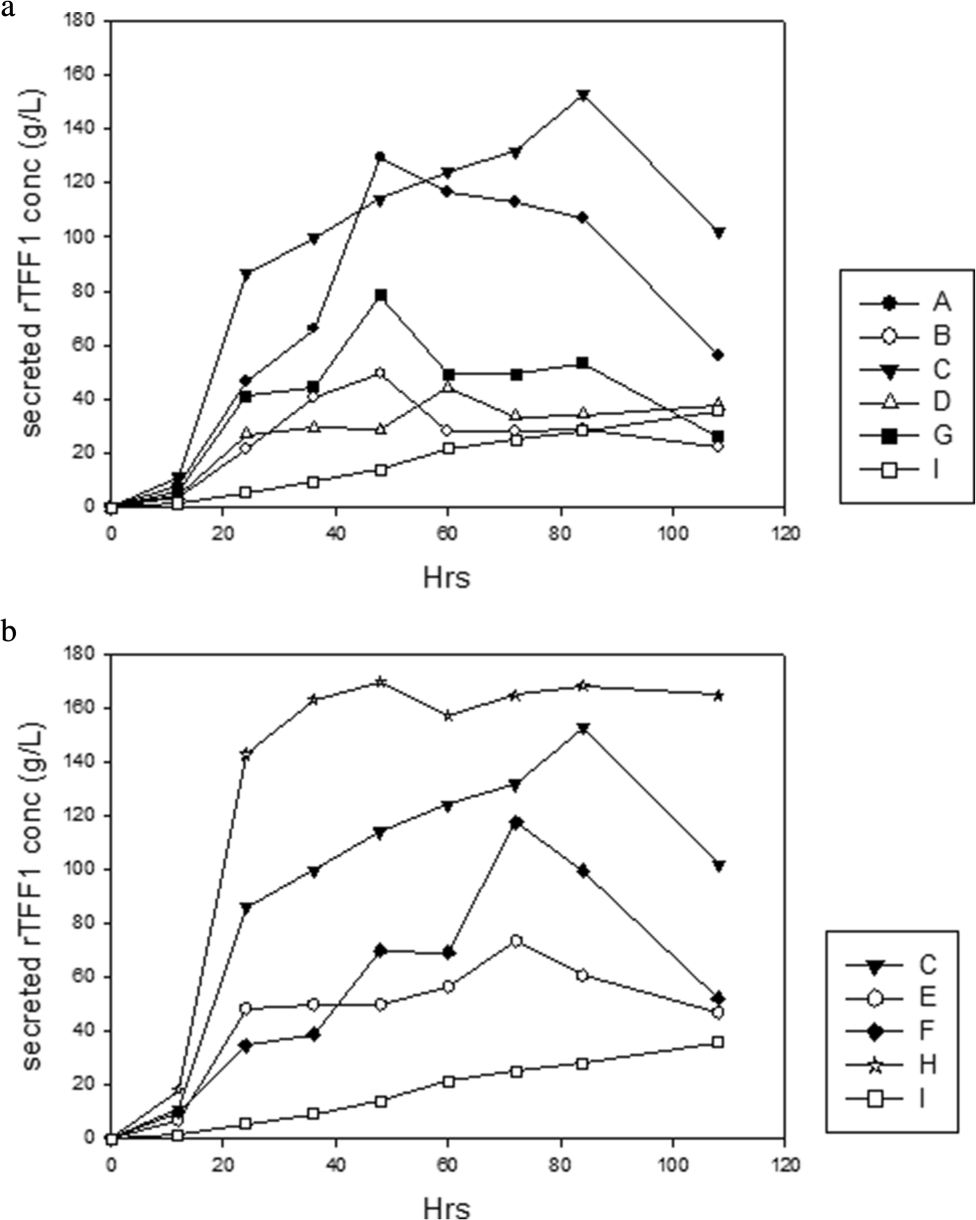
The protein yields of secreted recombinant TFF1 protein by various culture media. **(a)** effect of metal ions and nitrogen sources; and **(b)** effect of carbon sources. The cultures were taken at time intervals and the OD_600_ were measured. The productivity of rTFF1 was estimated by comparing the density of rTFF1 bands to standard rTFF1 on SDS-PAGE gels analyzed by Gel-Pro Analyzer^TM^ version 3.0 (Total-Integra Technology Co., Ltd, Taipei, Taiwan).

To enhance the industrial application of rTFF1, production of rTFF1 was improved by various media. Supplementation with Mn^2+^ to replace the Ca^2+^ elevated the rTFF1 productivity in trypton-based media (Broths A/I, and B/G), but not in soytone-based medium (Broth C/A) (Figure 6a). The results indicated that the nitrogen source was more effective than metal ions in rTFF1 productivity. Among nitrogen sources, soytone-based media (Broths A, C and I) were better than tryptone-based medium (Broths B, D and G) to enhance rTFF1 productivity; extra yeast extract improved the rTFF1 productivity in soytone-based medium but not in tryptone-based medium (Figure 6a). Various carbon sources were tested; among the tested Broths C/E/F/H/I, Broth H achieved the highest secretion productivity of rTFF1 (170 mg/L) at 48 hr (Figure 6b).

## Discussion

In this study, human TFF1 sequence was synthesized and cloned into expressing vector pET-TFF1 in which the C-terminal of rTFF1 contained His•Tag. In the *B. choshinensis* expressing vector pNCMO2-TFF1, the rTFF1 was constructed in which Sec signal peptide was fused in front of the rTFF1 and His•Tag sequence was in the C-terminal of rTFF1. These constructions were predicted to express functional rTFF1 of molecular mass 7.6 kDa and 7.7 kDa in cytoplasm and extracellularly, respectively. These two vectors successfully expressed rTFF1 in the cytoplasm of *E. coli* and secreted rTFF1 extracellularly by *B. choshinensis*, respectively (Figures 1 and 2). In the *E. coli* system, the pET-TFF1 expression vector was driven by the T7 promoter under IPTG induction, and rTFF1 was observed after two hours into the induction. The highest yield (21 mg/L) of rTFF1 was achieved 10 hours after induction, but the rTFF1 was unstable and degraded into lower molecular weight fragments after 34 hours of induction. In the *B. choshinensis* system, continuous expression of the pNCMO2-TFF1 vector was driven by the P2 promoter, and secreted by SP_Sec_ signal peptide. After cleavage of the signal peptide during the translocation process, the secreted rTFF1 was observed at 12 hours in the culture medium fraction. The highest yield (36 mg/L) of rTFF1 was achieved after 108 hours in the culture extracellular fraction, higher than the *E. coli* system of this study and previously reported levels ranging from 3.5 mg/L to 20 mg/L by *E. coli* [14] and other systems [15,16,20]. In this study, the optimal expression condition of rTFF1 by the *B. choshinensis* system was examined. The extracellular expression was expected to be more efficient for industrial scale-up production [17-19].

The 3D structure of the recombinant human TFF1 from *E. coli* was estimated by NMR spectra previously. A short α-helix packed against a two-stranded antiparallel β-sheet and three closely-packed loop structure was determined [20]. In another study, the structure of the disulfide-linked homodimer of human TFF1 was indicated [12]. In our expression system, the monomer, dimer, trimer, and tetramer forms of rTFF1s were observed on SDS-PAGE gel (Figure 3). The percentage of monomer, dimer, trimer, and tetramer rTFF1s from *E. coli* and *B. choshinensis* systems were about 45%, 32%, 15%, and 9% on SDS-PAGE gel, respectively. Using glutaraldehyde as a crosslinker can detect the existence of the correct folding and inter-disulfide bonds cross-linking between free thiol of rTFF1 or the interactions of mere proximity of rTFF1.

To maintain a gel-like structure in stomach, TFF1s are associated with mucins through ionic interaction, hydrophobic interaction and hydrogen bonds; Ca^2+^ ions enhance interaction [9,21,22]. The extraction from normal human gastric mucosa contained monomer, dimer, and complexes of TFF1 and were examined to be about 12-40%, 1-4%, and 57-83%, respectively [10]. In our study, rTFF1/EC was observed in four forms: tetramer (21.7%), trimer (23.6%), dimer (25.9%) and monomer (28.8%). The percentage was changed into about 23.8%, 24.3%, 24.4% and 27.5% in tetramer, trimer, dimer, and monomer forms after pH2.4 buffer treatment. The rTFF1/BC was observed as four forms tetramer (8.2%), trimer (21.7%), dimer (27.4%), and monomer (42.7%). However, after the pH 2.4 buffer treatment, the percentage was shifted into tetramer (13.5%), trimer (22.8%), dimer (29.2%), and monomer (34.5%). The rTFF1/EC and rTFF1/BC were highlighted in native gel (Figure 3). The more polymerization forms of rTFF1 expressed by our expression systems were similar to native status of human gastric mucus than other expression systems reported previously. The N-terminal of rTFF1/EC containing extra Met residues showed more polymer forms compared to rTFF1/BC. The rTFF1/BC was secreted extracellularly, the signal peptide was cleaved and extra Ala-Gly-Ser residues were left in the N-terminal. On native PAGE gel, the rTFF1/EC exhibited more mobility shifting than rTFF1/BC (Figure 3). The molecular size of rTFF1 was confirmed by MALDI-TOF spectrometry. The rTFF1s in different buffers (pH 7.0, pH 2.4) were reduced with or without DTT treatment. The similar molecular weights of rTFF1/EC and rTFF1/BC had monomer, dimer, trimer, and tetramer forms, with a trace amount of pentamer form observed in rTFF1/EC (Sup. 1)*.* The mobility shift between TFF1/EC and TFF1/BC on native gel was slightly different. Reducing treatment with DTT exhibited monomer forms for all the rTFF1s. However, different mobility shift, differences between polymeric forms of rTFF1/EC and rTFF1/BC on native PAGE gel and the unexpected mass increase of rTFF1/BC all indicated that other factors may contribute to the observed polymerization difference. It is possible that post-translational modification can occur on rTFF1/BC. Results of LC-MS/MS spectrometry and glycoprotein stained (Figure 4) showed that the glycosylation modification was added to the Ser residue on the N-terminal of rTFF1/BC*.*

The interaction between TFFs and mucins were pH-dependent, in which viscous response was stronger in buffer pH 2.0 than in buffer pH 7.4 [23]. The MUC5AC mucins are secreted from HT-29 cells and the disulfide linkage would undergo reduction at high a pH value (pH 8.0) [24]. It was suggested that the hydrophobic cleft which contained conserved amino acid residues, Phe^19^, Pro^20^, Pro^42^ and Trp^45^, between loop 2 and loop 3 of TFF1, provided a binding site for the ribose ring or an aromatic ring of amino acids. This intermolecular interaction would be formed with ligands or receptors [21]. In low pH conditions, the structure of TFF1 was more stacked through three pairs of disulfide bonds and this conformation may be more prone to protein-protein interaction through hydrophobic cleft interaction and hydrogen bonds. As seen in rTFF1 treated with DTT in pH 2.4 or 7.0 buffers, only monomer forms were present on native gel (Figure 3).

The concentration of total monomeric, dimeric and complex TFF1 from normal human gastric antral mucosa is about 2.8 to 13 μg/mg ,which is approximately 0.2-1% of total mucosal proteins [9]. In wound healing assays, added TFFs could promote cell migration but there was no effect on cell proliferation [10,25-27]. In cell line assays, the concentration of TFFs was about 7.5 nM to 0.24 μM levels that had an effect on *in vitro* wound healing assays [11,26,28]. The effective concentration of *in vivo* wound healing assay was about 7.5 nmol/kg [10]. In this study, the concentrations of 30 nM rTFF1 in medium had an effect on cell migration. The rTFF1/BC showed more promoted moving after pH 2.4 buffer pretreatment than the pH 7.0 buffer treatment rTFF1 (Figure 5a). On the other hand, the rTFF1/EC showed less capability of wound healing and did not exhibit enhancing capability after the pH 2.4 buffer pretreatment (Figure 5b). The extra N-terminal sequence of rTFF1/BC was not involved in the trefoil factor functional domain, but glycosylation modification might enhance the interaction of cell surface receptors or mucins with rTFF1, as compared to rTFF1/EC*.* The rTFF1/BC would likely be prone to associate to form more polymeric rTFF1 after the pH 2.4 treatment, and more efficiency interact with surface receptors or mucins. Therefore, a better wound healing capacity of rTFF1/BC was achieved than that of the rTFF1/EC*.* The rTFF1/BC in this study was expected to be more effective in stomach acidic conditions. Previously, glycosylated TFF2 in the stomach is the only TFF which had reported *in vivo* functional significance [29]. The O-linked serine rich glycoprotein was suggested to play a role in bacterial pathogenesis adhesion [30]. In this study, O-linked glycosylated rTFF1 exhibited better wound healing capacity which is a novel finding. To our knowledge, this is also the first report concerning O-linked glycoprotein which was secreted extracellularly by *Bacillus* or *Brevibacillus* species.

To improve rTFF1 secretion productivity, various media were tested to optimize yields. All the growth curves of *B. choshinensis* transformant cultured by various media were similar. In Figure 6a, the effects of metal ions and nitrogen sources were examined. It has been reported that metal ions play an important role in maintaining cell formation, metabolism and enzyme production and stability [31-34]. Supplements of Mn^2+^ instead of Ca^2+^ elevated rTFF1 protein secretion production only in the soytone-based medium (Broth A and I); supplements of the extra yeast extract (Broth C) covered the effect of metal ions. In the trypton-based medium, the effect of ions weren’t significant. The Broth G supplement with extra yeast extract showed better productivity at 48 hrs of culturing. Results suggested that a nitrogen source was more effective than metal ions in rTFF1 productivity. Among nitrogen sources, soytone based media (Broths A, C and I) were better than tryptone-based media (Broths B, D and G) in improving rTFF1 productivity; the extra yeast extract (15 g/L) improved rTFF1 productivity (Broth C at 84 hrs and Broth G at 24 hrs of culturing) (Figure 6a). It is a safer and more effective choice of soytone as a nitrogen source to avoid undesirable contaminants such as prions from animal nitrogen sources. Therefore, a medium composed of plant resource soytone and 15 g/L yeast extract was used to examine the effect of carbon sources. Figure 6b shows that sucrose, soluble starch, and glucose did not affect the growth of *B. choshinensis* transformant but affected the secretion of rTFF1 at various levels and time points. Broth H with higher glucose content (50 g/L) achieved the highest secretion productivity of rTFF1 (170 mg/L) at 48 hr of culturing; enhanced productivity and less culture time to achieve optimal production were both achieved.

## Conclusion

High levels extracellular production of recombinant human trefoil factor 1 (rTFF1) were achieved by *B. choshinensis,* superior to that of *E. coli*. The rTFF1 purified from *B. choshinensis* was unexpectedly glycosylated at the N-terminal serine residue and enhanced wound healing capacity. The rTFF1 purified from *B. choshinensis* also promoted better wound healing capacity after the pH 2.4 buffer treatment; this character suggests effective application potency in stomach acid conditions. The extracellular production by the *B. choshinensis* system was suggested to facilitate industrial production as well as ease of downstream processing. Production of active eukaryotic proteins through bacterial expression systems still remains “an art” [35]. This study provides a potent tool for producing recombinant human trefoil factor 1 to apply to gastric injury damage protection and wound healing.

## Methods

### Bacterial strains, plasmid and cell line

Strains of *E. coli* BL21 (DE3) (Novagen, Darmstadt, Germany) and *B. choshinensis* SP3 (TaKaRa, Shiga, Japan) were used for protein expression. *E. coli* JM109 (Promega, Wisconsin, USA) was used for DNA manipulation (Table 1).

**Table 1:** **The strains, plasmids, cell lines and primers used in this study**

**Strains, plasmids, cell lines and primers**	**Relevant characteristics**	**Source**
**Strains**		
*Escherichia coli* JM109	*end*A1 *rec*A1 *gry*A96 *thi hsd*R17 (*rK*-, *mK*+) *rel*A1 *sup*E44 (*lac*-*pro*AB) (*tra*D36 *pro*AB *lac* Z △M15, used as DNA manipulation strain.	Promega Co. (Wisconsin, USA)
*E. coli* BL21(DE3)	F^−^ *ompT hsdSB*(*rB*-*mB*-)*gal dcm*(DE3), used as protein expression strain.	Novagen (Merk, Darmstadt, Germany)
*Brevibacillus choshinensis* SP3	A highly safe host. *imp emp* and genes relating to sporulation have been disrupted. Used as protein expression strain.	TaKaRa Bio Inc. (Shiga, Japan)
**Plasmids**		
pET-29a(+)	Km^r^, a vector for *E. coli*	Novagen (Merk, Darmstadt, Germany)
pET-TFF1	Km^r^, pET-29a(+) (*Nde*I/*Hin*dIII::TFF1-TagHis6)	This study
pNCMO2	Nm^r^ and Amp^r^, a shuttle vector for *Brevibacillus* and *E. coli*	TaKaRa Bio Inc. (Shiga, Japan)
pNCMO2-TFF1	Nm^r^ and Amp^r^, pNCMO2 (*Bam*HI/*Hin*dIII::TFF1-TagHis6)	This study
**cell line**		
AGS	Human gastric adenocarcinoma	Bioresource Collection and Research Center, BCRC 60102 (Hsinchu, Taiwan)
**Primers**	**sequence (5 3′)** ^*^	
TFF1F4	GTTATA**CATATG**GAAGCTCAAACAGAAACATGTACAGTTG	This study
TFF1R2	GTTATA**AAGCTT**TTATTAATGATGATGATGATGATGAAATT	This study
PNCMO2F	CG**GGATCC**GAAGCTCAAACAGAAACATGT	This study
PNCMO2R	CC**CAAGCTT**TTAATGATGATGATGATGATGAAA	This study

^*^Restriction sites are in bold (CATATG for *Nde*I, AAGCTT for *Hind*III, and GGATCC for *Bam*HI).

### Construction of protein expression vectors and transformants

The gene *rtff1* of Human TFF1 was synthesized and cloned in PUC57-TFF1 vector by Quantum Biotechnology Inc. (Taipei, Taiwan, ROC). The novel *rtff1* gene was designed according to the preferred codons of *Bacillus subtilis* and can be found in a patent (Invention No. 472615, R.O.C.). The protein expression vectors pET-TFF1 and pNCMO2-TFF1 were derived from pET-29a(+) and pNCMO2, respectively. To generate pET-TFF1 and pNCMO2-TFF1, primer pairs were used (Table 1) and amplified by PCR. PCR reactions were preceded by 30 cycles (95°C, 30 s; 60°C, 30 s; 72°C, 1 min) followed by a 7 min extension at 72°C, the reaction mixtures containing 10 ng template DNA (PUC57-TFF1), 0.32 μM primer (each), 0.2 mM dNTP (each), and 1.25U of *Pfu* polymerase (Fermentas, Waltham, Massachusetts, USA) to obtain the fragment of *rtff1*. The PCR amplified product of TFF1 and pET-29a(+) were digested with *Nde* I and *Hind* III (Promega, Wisconsin, USA) respectively, and ligated (NEB, Hillsborough, USA) to obtain pET-TFF1, and then electro-transformed into *E. coli* JM109 to manipulate the plasmid. The desired plasmid was checked and electro-transformed into *E. coli* BL21(DE3) and checked again to obtain the desired transformant. A similar protocol was used to construct pNCMO2-TFF1. PCR reaction was carried out over 35 cycles (95°C, 30s; 60°C, 30s; 72°C, 1 min) followed by a 10 min extension at 72°C in 50 μl reaction buffer. The PCR amplified *rtff1* and pNCMO2 were digested with *Bam*HI and *Hind* III (Promega, Wilconsin, USA), and then ligated and electro-transformed into *E. coli* JM109 to manipulate pNCMO2-TFF1. The desired plasmid was transformed into *B. choshinensis* SP3 with the Tris/PEG method as described (Cat. #HB100, TaKaRa, Shiga, Japan). All constructs were confirmed by restrict enzyme digestion and DNA sequencing (Sequence Center, National Chung Hsing University, Taichung, Taiwan).

### Protein expression and purification

To express protein, transformant *E. coli* BL21(DE3) (pET-TFF1) were cultivated at 37°C in 2 × TY medium (tryptone 16 g/L, yeast extract 10 g/L, NaCl 5 g/L) supplement with 30 μg/mL kanamycin. At OD_600_ about 1.0, a final concentration of 1 mM IPTG was added into the medium and then cultured for 4 hours. Transformant *B. choshinensis* (pNCMO2-TFF1) was cultivated at 30°C in 2SY medium (glucose 20 g/L, soytone 40 g/L, yeast extract 5 g/L, CaCl_2_.2H_2_O 0.15 g/L) supplemented with 50 μg/mL neomycin with 120 rpm shaking for 5 days. *E. coli* BL21(DE3) (pET-TFF1) cells were harvested by centrifugation and suspended in lysis buffer (20 mM Tris–HCl, pH 8.0, 10% glycerol, 5 mM phenylmethylsulfonyl fluoride, and 5 mM β-mercaptoethanol). The cell suspension was disrupted by ultrasonic treatment, and the extract was clarified by centrifugation at 13,000 × g for 20 min. The supernatant was mixed with Ni-NTA affinity resin (GE Healthcare) for further purification. Transformant *B. choshinensis* (pNCMO2-TFF1) cells were centrifuged and removed; the supernatant was treated with 60% ammonium sulfate to precipitate the secreted proteins. The precipitated proteins were separated by centrifugation at 10,000 × g for 20 min, resuspended in 10 mL Tris (10 mM, pH 8.0) and dialyzed against the same buffer (10 m M Tris, pH 8.0). The protein suspensions were then mixed with Ni-NTA affinity resin (GE Healthcare) for further purification. Both mixtures of Ni-NTA affinity resin and proteins were gently agitated in the presence of 10 mM imidazole for 2 h at 4°C. The mixed resin was then washed repeatedly with wash buffer (10 mM K_2_HPO_4_- KH_2_PO_4_, pH7.0, 20 mM imidazole), and then eluted with 250 mM imidazole-containing wash buffer. The concentration of eluted protein was determined by the Bradford method using bovine serum albumin (BSA) as standard [36].

### Culture conditions, measurement of cell growth and optimization of fermentation culture

Various culture media containing various carbon sources (glucose, sucrose or soluble starch) and various nitrogen sources (soytone or tryptone) were used to optimize the protein secretion production. Culture media were incubated in baffled shake flasks at 30°C for 120 rpm. The tested media were listed in Table 2.

**Table 2:** **The composition of the media tested in this study**

**Medium**	**Composition**		
	**Carbon source**	**Nitrogen source**	**Metal ions**
A	20 g/L glucose	40g/L soytone	0.012 g/L MnCl_2_
		5 g/L yeast extract	
B	20 g/L glucose,	40 g/L tryptone	0.012 g/L MnCl_2_
		15 g/L yeast extract	
C	20 g/L glucose	40 g/L soytone	0.15 g/L CaCl_2_
		15 g/L yeast extract	
D	20 g/L glucose	40 g/L tryptone	0.15 g/L CaCl_2_
		5 g/L yeast extract	
E	20 g/L soluble starch	40 g/L soytone	0.15 g/L CaCl_2_
		5 g/L yeast extract	
F	20 g/L sucrose	40 g/L soytone	0.15 g/L CaCl_2_;
		5 g/L yeast extract	
G	20 g/L glucose	40 g/L tryptone	0.15 g/L CaCl_2_
		15 g/L yeast extract	
H	50 g/L glucose	40 g/L soytone	0.15 g/L CaCl_2_
		15 g/L yeast extract	
I	20 g/L glucose	40 g/L soytone	0.15 g/L CaCl_2_
		5 g/L yeast extract	

### Tricine-SDS-PAGE and Western Blot analysis

The rTFF1 produced by *E. coli* BL21(DE3) (pET-TFF1) and *B. choshinensis* (pNCMO2-TFF1) at various time expression intervals were analyzed by Tricine-SDS-PAGE [37]. The yield of rTFF1 was estimated by comparing the density of the rTFF1 bands to standards (serial dilutions of 103 mg/L purified rTFF1) on SDS-PAGE gels analyzed using Gel-Pro Analyzer^TM^ version 3.0 (Total-Integra Technology Co., Ltd, Taipei, Taiwan). The electrophoresis gel was then transferred into PVDF membranes (Millipore, Darmstadt, Germany). The mouse anti- His•tag (Millipore, Darmstadt, Germany) and goat anti-mouse HRP (Millipore, Darmstadt, Germany) were used to immunize the rTFF1. The presentations of rTFF1s were then stained with Western Lightning^TM^ Plus-ECL (Perkin Elmer, Inc., USA), analyzed by Fusion-Capt advance analysis software (Vilber Lourmat, France).

### Determination of homodimer and polymeric forms of rTFF1

To examine the existence of dimer forms, the proteins (0.103 mg/mL) were treated with 0.05% glutaraldehyde at room temperature for 1 h and then were analyzed with 16.5% Tricine-SDS-PAGE. To examine the rTFF1 polymeric forms, the proteins were treated with phosphate buffer (pH 7.0) and Na_2_HPO_4_-citric acid buffer (pH 2.4) [38], and then analyzed by 14% native PAGE [39]. For comparison, 10 mM dithiothreitol (DTT) was added as a control and proteins were stained with silver stain kits (Pierce, USA).

### Wound-healing analysis

AGS cells were maintained in Ham’s F-12 K Medium (1.802 g/L glucose, 146 mg/L glutamine) containing 10% FBS, 100 U/mL Penicillin, 0.25 μg/mL Amphotericin B, and 100 μg/mL Streptomycin (Gibco, Germany). The cells were incubated at 37°C in an atmosphere of 5% CO_2_. The confluent cultures were washed with Ham’s F-12 K Medium (serum free), and the cells (5.0 × 10^4^) were loaded into culture inserts (ibidi, Germany) within culture dishes (35 mm × 10 mm) which incubated in Ham’s F-12 K Medium containing 10% FBS. After 18 h of culturing, culture inserts were removed and various concentrations of rTFF1s were added and cultures were maintained in Ham’s F-12 K Medium (with or without 10% FBS) and incubated at 37°C in an atmosphere of 5% CO_2_. Photographs were made approximately every 24 h after starting at 0 h (at that time, rTFF1 had not been added). The complete experiment was done in 48 h; wound closure was monitored and photographed at 0, 24 and 48 h with a Nikon TS100 inverted-microscope and a Nikon D5100 camera (Nikon, Japan).

### Mass spectrometry assay

To examine the existence of polymeric forms, the purified rTFF1/ECBL21(DE3) (pET-TFF1) and *B. choshinensis* (pNCMO2-TFF1) were confirmed by matrix-assisted laser desorption/ionization mass spectrometry (MALDI-TOF) (Voyager-DE Pro, Applied Biosystems, USA) (Biotechnology Center, National Chung Hsing University, Taichung, Taiwan). Additionally, the modification of rTFF1 was also confirmed by LC-MS/MS (QSTAR®XL, Applied Biosystems, USA).

### Glycosylation assay

To advance the determination of post-translational modification of rTFF1, the protein from *B. choshinensis* (pNCMO2-TFF1) was confirmed with a glycoprotein stain kit (Pierce, USA).

### Statistical analysis

The results were expressed as the mean ± SD of data obtained from quadruplicate experiments. The statistical significance was estimated using a SPSS 12.0 Tukey HSD. Values with *p* < 0.05 were considered significant.

## Additional file

Additional file 1: Figure S1. The polymeric forms analysis of rTFF1 purified from *B. choshinensis* (pNCMO2-TFF1) or BL21(DE3) (pET-TFF1) by MALDI-TOF spectrometry. (a) various buffers; and (b) various buffers and DTT. The rTFF1 proteins purified from *B. choshinensis* (pNCMO2-TFF1) and BL21(DE3) (pET-TFF1). The purified rTFF1s were then analyzed by MALDI-TOF spectrometry. The TFF1/EC 7.0 and TFF1/EC 2.4 represented the rTFF1 proteins purified from BL21(DE3) (pET-TFF1) dialyzed against PBS buffer (50 mM Na_2_HPO_4_-NaH2PO_4_, pH 7.0) or Na_2_HPO_4_-citric acid buffer (pH 2.4), respectively. The TFF1/BC 7.0 and TFF1/BC 2.4 represent the rTFF1 proteins purified from *B. choshinensis* (pNCMO2-TFF1) and dialyzed against PBS buffer (50 mM Na_2_HPO_4_-NaH2PO_4_, pH 7.0) or Na_2_HPO_4_-citric acid buffer (pH 2.4), respectively. The TFF1/EC 7.0-DTT, TFF1/EC 2.4-DTT, TFF1/BC 7.0-DTT and TFF1/BC 2.4-DTT represented the rTFF1 proteins purified from BL21(DE3) (pET-TFF1) or *B. choshinensis* (pNCMO2-TFF1) dialyzed against PBS buffer (50 mM Na_2_HPO_4_-NaH2PO_4_, pH 7.0) or Na_2_HPO_4_-citric acid buffer (pH 2.4) supplemented with 10 mM Dithiothreitol (DTT), respectively. The numbers indicate: (1) monomer; (2) dimer; (3) trimer; (4) tetramer; and (5) pentamer forms.

## Abbreviations

rTFF1: Recombinant trefoil factor I
rTFF1/EC: Purified recombinant trefoil factor I produced by *Escherichia coli*
rTFF1/BC: Purified recombinant trefoil factor I produced by *Brevebacillus choshinensis*
