# Supplementary figures and images for: Functional expression of recombinant human trefoil factor 1 by Escherichia coli and Brevibacillus choshinensis
Source: BMC Biotechnol. 2015 May 20;15:32. doi: 10.1186/s12896-015-0149-5 (PMC4438461; doi:10.1186/s12896-015-0149-5)

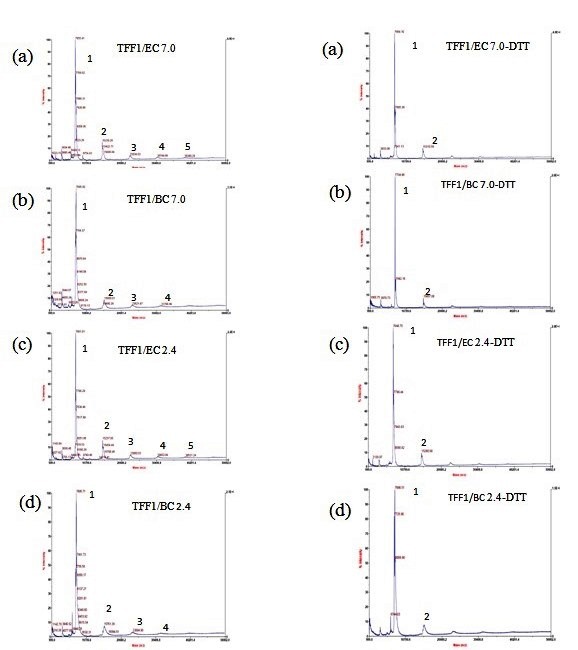

Supplement: Additional file 1: Figure S1. — The polymeric forms analysis of rTFF1 purified from B. choshinensis (pNCMO2-TFF1) or BL21(DE3) (pET-TFF1) by MALDI-TOF spectrometry. (a) various buffers; and (b) various buffers and DTT. The rTFF1 proteins purified from B. choshinensis (pNCMO2-TFF1) and BL21(DE3) (pET-TFF1). The purified rTFF1s were then analyzed by MALDI-TOF spectrometry. The TFF1/EC 7.0 and TFF1/EC 2.4 represented the rTFF1 proteins purified from BL21(DE3) (pET-TFF1) dialyzed against PBS buffer (50 mM Na2HPO4-NaH2PO4, pH 7.0) or Na2HPO4-citric acid buffer (pH 2.4), respectively. The TFF1/BC 7.0 and TFF1/BC 2.4 represent the rTFF1 proteins purified from B. choshinensis (pNCMO2-TFF1) and dialyzed against PBS buffer (50 mM Na2HPO4-NaH2PO4, pH 7.0) or Na2HPO4-citric acid buffer (pH 2.4), respectively. The TFF1/EC 7.0-DTT, TFF1/EC 2.4-DTT, TFF1/BC 7.0-DTT and TFF1/BC 2.4-DTT represented the rTFF1 proteins purified from BL21(DE3) (pET-TFF1) or B. choshinensis (pNCMO2-TFF1) dialyzed against PBS buffer (50 mM Na2HPO4-NaH2PO4, pH 7.0) or Na2HPO4-citric acid buffer (pH 2.4) supplemented with 10 mM Dithiothreitol (DTT), respectively. The numbers indicate: (1) monomer; (2) dimer; (3) trimer; (4) tetramer; and (5) pentamer forms. [file 12896_2015_149_MOESM1_ESM.jpeg]
